# Bibliographical Notices

**Published:** 1854-10

**Authors:** 


*Manual of Human Microscopical Anatomy.* By A. Kölliker, Professor of Anatomy and Physiology in Würsburg. *Translated by* George Busk, F. R. S., and Morris Huxley, F. R. S. *Edited, with Notes and Additions*, by J. Da Costa, M. D. *Illustrated by three hundred and thirteen engravings on wood.* Philadelphia: Lippincott, Grambo & Co., 1854.

The operations of nature differ from those of man in this—that while the works of the latter are open and apparent to us all, the former performs her processes so secretly and by movements so minute and gradual, as to place them entirely beyond the sphere of our vision. And thus invisible must they always have remained to us without the aid of the microscope. By its assistance, however, we have been enabled to enter nature's secret laboratory, her inmost sanctum, and there witness "the very pulse of the machine." No discovery has been more prolific in the results it has afforded. It has greatly extended the boundaries of science, exhibited myriads of existences unknown before, and given us a new revelation of the infinite power and majesty of the Almighty.

Histology, the science which treats of the minute structure of all animal and vegetable organisms, may be said to have had its birth, growth, and attained its present maturity within the last thirty years. Previous to that period it was merely "a disjointed collection of unconnected observations." It became a science, in fact, only in 1838, when Schwann discovered the origin of all organic tissues from a common centre—the cell.

Since that period numerous and zealous cultivators of the subject have appeared, among whom the author of the above treatise, Prof. Kölliker, stands deservedly high. In evidence of the estimation in which he is held abroad, we need only mention, that from among the great number of admirable foreign works lately published, the above manual of human microscopical anatomy has been deemed most worthy of selection for translation by the Sydenham Society. This just ard fitting tribute to the high endowments of its author, should, in itself, be sufficient to stamp its value to the profession.

It is known to many of our readers that upwards of a year ago, Messrs. Lippincott, Grambo & Co., of this city, announced their intention of issuing a translation of this work. The American editor, to whom the translation was entrusted, had performed more than half of his laborious task, expecting to finish the remainder in about a year, when he first "learned that the Sydenham Society intended to publish an English edition of the work, and that indeed, the first volume of this was ready to be issued. As the time and labor requisite for an original translation would have prolonged the publication of the work far beyond that of the Sydenham Society, the editor did not judge it expedient to delay the appearance of an American edition longer than was necessary. He resolved, therefore, to adopt the translation of the Sydenham Society, and merely to append such notes as he intended to have added to his own edition." We need not say that we greatly regret this unfortunate contre-temps, as we should have been much pleased to have had an American translation of this great work.

Prof. Kölliker makes two divisions of his subject, General Histology and Special Histology. These are prefaced by an introduction, in which the past history of the science, its present position, and the different aids required in studying it, are briefly mentioned. Under the head of General Histology the author treats of, 1st, the elementary parts, simple and compound; and 2d, of the tissues, organs, and systems which are composed of these elementary parts, in obedience to determinate laws. The most important of the simple elementary parts "are the cells, which not only form the starting point of every animal and vegetable organism, but also, either as cells, or after having undergone manifold metamorphoses, make up the body of the perfect animal, and in the simplest animal and vegetable formations (unicellular animals and plants,) even enjoy an independent existence." Thirty pages are devoted to the description of their composition, form, chemical relation, multiplication by division, &c. Under the latter head, the author remarks:

"*A multiplication of cells by division* certainly takes place in the red blood-corpuscles of the embryos of Birds and Mammalia, and in the first colorless blood-corpuscles of the Tadpole (Remak). It takes place also, in all probability, in the colorless blood-corpuscles of embryos, and in the chyle-corpuscles of adult Mammalia under certain circumstances. In all these cases, we see, in elongating cells, the production of two nuclei from the originally simple nucleus, apparently by division; the cells then suffer constriction in the middle, and contract more and more around the nuclei, as they recede from each other, and at last separate into two cells, each of which contains a nucleus. In the chick we see blood-corpuscles in all conceivable stages of separation, so that at length they are connected only by a delicate thread, and there can be no doubt whatever as to the actual occurrence of this process.

Whether division ever takes place in other cells than these is not yet determined. If it be allowable to explain, by this process of division, the occurrence of constricted cells with two nuclei, we may suppose it to take place in the nerve-cells, which, in young mammalia, are not unfrequently more or less divided or even united merely by a narrow isthmus, and also in the ciliated epithelium cells, which, although rarely, present two or three enlargements lying one behind the other, each with a nucleus. A peculiar kind of cell-development, which is very closely related to division, occurs in the formative cells of the ivory, which as they go on growing, multiply their nuclei and become constricted from time to time, so that whilst the portion next the ivory ossifies, the other serves in a manner as a reserve for the subsequent formation of fresh ossifying tissue."

The description of the different tissues, epidermic, cartilaginous, elastic, connective, osseous, smooth, muscular, transversely striated, nervous tissue of the blood-vascular glands, and the tissue of the true glands, concludes the subject of General Histology. For our author's views on the development of the two forms of connective tissue which materially differ from those which Virchow holds, we refer our readers to pages 85 and 94.

Regarding "Smooth muscular tissue," a special study of the author, a number of interesting remarks are made.

Under the division of "Special Histology," the external integument; the muscular system; the osseous system; the nervous system; the digestive organs; the respiratory organs; the urinary organs; the supra renal glands; the sexual organs; the vascular and the higher organs of sense, are minutely and accurately described. We may mention here, that at the conclusion of each subject throughout his work the author appends a full account of the methods to be employed in its investigations, and a copious bibliographical list of references. This adds very much to the value of the work for students, saving them much time and trouble. Interesting physiological and pathological remarks, where the subject demands it, are also added.

In the extracts we shall make from this division of the work, we shall mainly indicate those points in which originality is claimed by the author.

THE SKIN. On the subject of nuclei in the fat-cells of the adult, he makes the following remarks :—

The nuclei in the fat-cells of the adult have not, as far as I am aware, yet been observed, excepting by Bendz (Almind. "Anat." p. 122, tab. I. fig. 4), who rarely, very rarely, noticed even two pale nuclei with nucleoli. It is true that Mulder (p. 601), states that they are furnished with one, rarely with two, nuclei, but Donders and Moleschott (*ib.*, p. 602, *et seq.*), upon whom Mulder seemes principally to rely, expressly say that they did not detect the nuclei; nor does Donders (in the "Holländ. Beitr.," I. pp. 57, 61), say anything about nuclei. I *invariably* find them when the fat has partially disappeared from the cell. In cells completely filled, I first distinctly noticed them, in some cases in the marrow, and in the fat-cells in the muscles; but I do not hesitate in the least to affirm their constant occurrence in all fat-cells, since no one can suppose that they are not formed until after the disappearance of the fatty contents."

For the author's anatomical and physiological views on "The tactile papiliæ," to which he has devoted much attention, we must refer our readers to the work itself, the article being entirely too long for our pages. We shall merely extract the following :—

"E. H. Weber has endeavored to demonstrate, in his last able Treatise upon the sense of touch, that it is only the termination of the nerves in the skin, not the fibres in the nervous trunks, which are the mediators of the sensations of pressure, warmth, and cold; and he has expressed a supposition, that tactile organs as yet unknown may exist in the skin. R. Wagner believes that he has, in fact, found these organs in his so-called *corpuscula tactûs*, and he supposes that, composed as they are, according to him, of superimposed membranes, in the interspaces of which a very minute quantity of fluid is lodged, like elastic cushions or a bladder filled with water, they are specially fitted to receive impressions from the epidermis at the extremity which is directed towards it, and to transmit them to the ends of the nerves which lie in and upon them."

"In my opinion, Weber's view of the greater sensibility of the terminations of the nerves in the skin, can hardly be doubted; but, on the other hand, there is no obvious reason, *á priori*, why peculiar hitherto unknown organs should exist to that end; nor why the condition to which we have already referred, the more *isolated course* of the fibres of the nervous tubules in the papillæ and terminal plexuses, their fineness, *superficial position*, and the *delicacy* or *absence* of the *neurilemma*, may not abundantly suffice as an explanation. That Wagner's so-called *corpuscula tactûs*, my axile corpuscles, are not tactile organs in the sense intended by Weber, is easily demonstrable. Independently of the erroneousness of his views of their structure, and of the fact that the nerves are not distributed in them, but only proceed along them, outside, in order, in many cases, to terminate even beyond them—we find *that all the essential functions of the skin are also performed without such corpuscles.* The feeling of warmth and cold, of orgasm, of tickling, of pressure, of pricking, of burning, of pain, are found partly over the whole surface of skin, partly in places where these corpuscles are certainly absent, which sufficiently shows *that they have not in the remotest degree the signification ascribed to them by Wagner.* However, it is not likely that they exist for nothing in those particular localities, in which the sensibility to pressure is the greatest, and which we use especially as tactile organs, as the ends of the fingers, the point of the tongue, and the border of the lips; and I consider them as parts, *which in consequence of their being composed principally of dense, imperfectly-formed elastic tisssue, confer a certain solidity upon the points of the papillæ, and serve as a firm support for the nerves,* in consequence of which, a pressure, which in other situations is not sufficient to affect the nerve, here takes effect. They would, in fact, be organs, like the nails and phalangeal bones, not essential and indispensable to the sense of touch, but only conferring upon it a greater acuteness than elsewhere. If, in this sense, they are to be called *tactile corpuscles*, I have nothing to say against the term, but then the phalanges and the nails, the 'whiskers' of animals, &c., equally deserve the name of tactile organs."

The microscopist will find some highly interesting and useful remarks on the influence of various reagents on the epidermis on page 147, and on the blood globules on page 709.

Muscular System. Our author combats Bowman's views on the primitive particles, "Sarcous elements," of muscular fibre. On this point he remarks:—

"As regards the notion adopted by Bowman, Dobie, and others, that the fibrils are constituted of still more minute particles (sarcous elements), it may perhaps be stated, as the study of development shows, that the fibrils do, in fact, at first appear to consist of separate particles. But the question is, whether in the adult such elementary particles continue to be evident, and this, at present, I am inclined to deny."

On the course of the *primitive muscular fasciculi*, the editor incorporates into the text the following remarks, taken from the author's larger work:—

"It was formerly supposed that the *primitive muscular fasciculi* ran in a perfectly straight direction towards their insertions, without dividing or anastomosing; but this is not correct. Recent investigations, made partly alone, partly with the assistance of Dr. Corti, have demonstrated the anastomosis of the fasciculi of striped muscles in the hearts probably of all Mammalia. We observed anastomosing fasciculi in man, in the rabbit, dog, cat, calf, frog, heron, and Leydig has seen them in the ruff. In the Mammalia and in man, they are frequent and extremely delicate, and the anastomosis occurs by means of short transverse or oblique branches extending between parallel fasciculi."

The whole article on the muscular system is most excellent, and will well repay a careful perusal.

The description of the osseous system is very complete, comprising upwards of seventy pages. We would direct particular attention to the author's remarks on cartilage and its ossification. They differ very materially from those of Robin, who is charged by the author with having entirely misunderstood his statement.

The next subject treated of is the *Nervous System.* As most worthy of notice we invite the attention of students to his researches on the axis-cylinder, a mooted question among microscopists. His conclusions are as follows:

"These are the most important facts connected with the axis-cylinder. The conclusion which may be drawn from them, appears to me, to be simply this, that the axis-cylinder is not an artificial product, but that it must be regarded as an essential constituent of the living nerves. The only objection which can be urged against this opinion consists in the circumstance, that the axis-fibre cannot be seen in living fresh nerves, and that it cannot generally be distinguished, as a special structure, in the interior of the nerve-tubes without the aid of reagents. But it must be remarked that it *can also be brought into view in nerves that are still warm.* Thus I find well-marked projecting axis-fibres at the roots of the cerebral nerves in Frogs just kille l, which I have examined as quickly as possible, after the application of a solution of sugar, particularly in those of the optic, trigeminal, and *vagus*, also in the spinal nerves, for instance in the second. I see them under the same conditions in the peripheral nerves of the Frog that have been teased out, and, in these nerves, have on several occasions even distinctly noticed the axis-fibres in the form of convoluted filaments, in larger drops of the nerve-pulp expressed from the tubes."

Under the head of *the Digestive Organs*, we have a full description of the intestinal canal; of the liver; of the pancreas; and of the spleen. On the lingual papillæ, we have the following remarks:—

"The lingual papillæ present many varieties, the following of which are the most important: 1. The *papillæ filiformes are all elongated,*

and provided with very considerable epithelial processes. The appearance of what is commonly called a gastric furred tongue, depends principally upon the growth of the epithelial processes of the *papillæ filiformes*, which, all directed backwards and in close apposition, form apparently a peculiar white coating. If the processes become longer, so that the *papillæ filiformes* measure 1½–2 lines, we have the *lingua hirsuta or villosa*, which is not uncommon in various disorders; and at length forms may be produced, in which the tongue looks as if it were covered with hairs, 4–6 lines long. 2. The *papillæ filiformes possess very small epithelial processes, or none at all*, and are hardly distinguishable from the smaller *p. fungiformes*. 3. *The papillæ filiformes do not exist as special elevations, but are imbedded in a general epithelial investment of the dorsum of the tongue.* Tongues may be observed, particularly in old people, which, without being furred, in some spots or over a large extent, present no papillæ at all, but have either a perfectly smooth surface, or exhibit only a few linear elevations, corresponding with the rows of papillæ which would, otherwise, exist there. In these places we find the epithelium more developed, and beneath it small papillæ, more of the ordinary form. Tongues which, with better developed papillæ present a smooth surface, are again different from these; here the smooth or cracked surface is produced by the papillæ being glued together by superabundant epithelium, mucus, blood, pus-corpuscles, and mucedinous or yeast-like fungi. 4. *The epithelial processes of the filiform papillæ are covered with mucedinous fungi.*"

For his views on the *lacteals* we must refer to pages 513 and 515.

The structure of the liver is very fully described. As regards the commencement of the hepatic ducts, after showing that "The hepatic cells are so arranged in the islets as to form a *network* by the simple apposition of their flat surfaces, without the assistance of any foreign connecting intermediate substance or investing coat," he remarks:

"If, as results from the above analysis, the secreting parenchyma of the liver consists of a *solid network of hepatic cells*, one cannot but be struck with its great difference from all the other glands of the body; and the important question arises, how, with this arrangement, the secretion is conveyed from the interior of the cells, in which we suppose it to be formed, and finally carried away. Anatomy here gives no sufficient reply; for although the ramifications of the hepatic ducts have been traced accompanying the *vena portæ* as far as the hepatic islets, yet, as respects the connection of their finest twigs with the hepatic network of cells, no definite answer has been afforded; and indeed, up to the present time, no satisfactory account even of the structure of the former has been given. Without entering further, in this place, into the distribution of the hepatic ducts, I will only observe, that in carefully made microscopic preparations we not unusually find fragments of the finer and finest ducts, the *ductus interlobulares* of Kiernan, between the hepatic islets, and readily obtain evidence that they are constructed according to the ordinary type of excretory ducts.

Often as I have sought for a direct communication of the finest canals with the hepatic networks, I have not yet directly observed it; which is, indeed, by no means surprising, if we consider the softness of the parts with which we have to do; but unfortunately the result is a *hiatus* in the minute anatomy of the parts, which can hardly be made good by hypotheses. As such, however, I would offer the supposition, that the finest ducts impinge directly upon the columns of the network of the hepatic cells, as the diagram in Fig. 221 shows, so that their cavity is terminated by hepatic cells: and, judging from the scantiness of the finest branches of the hepatic duct, I believe that such communications exist, in no very great numbers, at the circumference of the hepatic islets.

Whatever view we may take of the connection of the hepatic cell-networks with the efferent biliary canals, it is undeniable that any such connection only takes place *upon the surface of the hepatic islets* and not in their interior, and that, therefore, the bile which is formed here, *must be transmitted outwards from cell to cell.* Such a process of transmission through closed cells, involves, as vegetable physiology teaches us, no impossibilities; but it can hardly take place so rapidly as in those localities where actual canals subserve this purpose."

Upon the import of the changes in the blood-corpuscles of the *Spleen*, whether they are physiological or pathological, he maintains their physiological nature, regarding their disintegration as a normal occurrence; see page 561.

We must pass over his description of the lungs; the thyroid gland; the thymus; the urinary organs; the supra-renal glands; and the sexual organs, though we find much in them that we would like to extract, to arrive at the eye, on many points of which structure he entertains novel views. Of the *retina* he says:—

"The *retina* is a delicate membrane; when recent, almost perfectly transparent and clear, and after death whitish and opaque. It commences at the point of entrance of the optic nerve, with which it is, in part, continuous. Its thickness at first is 0·1 of a line, but as it extends anteriorly it soon diminishes to 0·06, until ultimately, close to the anterior border of the *retina*, it is not more than 0·04 of a line in thickness, and finally terminates quite abruptly. Notwithstanding this various thickness, the following layers from without to within may be evidently distinguished in all parts of it; 1, the *layer of rods and cones* [bacillar layer]; 2, the *granular layer;* 3, the *layer of gray nerve-substance;* 4, the *expansion of the optic nerve;* and 5, the *limitary membrane.* These layers, with the exception of the innermost, which is of uniform thickness throughout, in general become thinner towards the front, in correspondence with the diminished thickness of the whole *retina.*"

These layers and their mutual connections being minutely described, he remarks :—

"In spite of the obscurity which, from what precedes, still hangs over a very important point in the anatomy of the *retina*, physiology may nevertheless even at present draw some useful conclusions from the facts in our possession. In the first place, since the demonstration by H. Müller and myself of its connection with the radiating fibre-system and the 'granules,' the bacillar layer appears in quite a different light from that in which it was previously held, and it is now obviously impossible to regard it, with Brucke, as a catoptric, reflecting apparatus. I look upon the 'rods' and 'cones,' which may also be said to correspond in all chemical characters with the nerve-fibres of the *retina*, and the whole of the radiating fibre-system of the *retina*, as *true nervous* elements; and venture at the same time to broach the bold supposition, founded upon a less established basis, which has been already thrown out, that the 'rods' and 'cones' are the *true percipients of light*, and that they communicate their condition to the fibres of the optic nerve, by means of the direct or indirect connection of their fibrous processes with the former, through which again the impressions are conveyed to the *sensorium.* That the optic-fibres in the nervous expansion of the retina, do not perceive light, appears to me to be proved by the circumstance : 1, that the point of the *retina*, where those fibres alone, and no other elements of the *retina*, are found, viz. at the entrance of the optic nerve, is not sensitive to light ; 2, that the optic fibres are *superimposed upon each other in such numbers*, in almost every part of the *retina*, and above all in the neighborhood of the *macula lutea*, that it is impossible that they should perceive light, inasmuch as each luminous impression, owing to the transparency of the fibres, must in any case always affect many of them, and consequently would of necessity give rise to confused sensations; and 3, because the part of the *retina* in which there is no continuous layer of nerve-fibres on the inner surface, that is to say, the 'yellow spot,' is the most sensitive to luminous impressions. Under this notion, the import of the 'rods,' and their remarkable arrangement, would be intelligible, and the almost inexplicable correspondence in the size of the images of the smallest distinguishable interspaces between two objects, with the diameter of the 'rods' and 'cones,' be placed in its true light. I consider it impossible to say anything with respect to the import of the other elements of the *retina*, for although the 'granules' may be compared to minute bipolar ganglion-corpuscles, and their continuity with the radiating fibres is known, this affords as little ground for discussion as to their function, as is given by the knowledge of the fact that the large nerve-cells of the inner layers have numerous processes and probably free terminations."

In the article on the ear, the editor acquaints us in a note with Prof. Kölliker's recent researches on the termination of the cochlear nerves. The anatomical description we are obliged to omit. He says :—

"Based upon these histological data, Kölliker advances some interesting and novel views with regard to the physiology of the cochlea. The existence of the peculiar nervous apparatus at the extremity of the cochlear nerves, renders it, he thinks, highly probable that this is the only portion of the nerve which possesses the power of receiving sonorous undulations. These undulations reach the cochlea through the fenestra ovalis, and are not communicated, as most physiologists suppose, by the osseous lamina to the cochlear nerve, since the latter lies free in the labyrinth and is unconnected with the osseous zone. The isolated position of the cochlear nerve, its movability and great softness, renders it, Kölliker further surmises, specially applicable to receive the undulations transmitted by the fluid of the labyrinth. Regarding the special function of the cochlea, he is inclined to attribute to it a greater physiological importance, than to any of the other parts of the labyrinth. The free position of the expanded portion of the nerve, and the extent of surface over which its numerous terminal fibres are spread, constitute it, he thinks, an organ of great delicacy of hearing, enabling us to distinguish several sounds at once, and also to estimate accurately their exact pitch. The ganglionic termination of the cochlear nerves, Kölliker concludes, establishes a surprising analogy between the auditory and visual apparatus, since the sensory nerves of both are possessed at their peripheral expansion of a ganglion, which receives the impressions, the nerve-fibres merely serving to conduct them to the brain. (*Vid.* Kölliker, "Ueber die Endigimgen des Nervus Cochleæ und di Functions der Schnecke," Wurzburg, 1854.)—DaC."]

Our limits have necessitated us to omit many passages we had marked for extract. What we have selected, will, however, suffice to impart some idea of the work. We may say in conclusion, that, throughout his Manual, the author displays not only a perfect acquaintance with the subject, but that there are few questions of either structure or function on which he has not thrown some light. As a text-book on Human Histology for the use of students and practitioners, we regard it, in fact, as unrivalled. For these reasons we strongly recommend it to such of the profession, as desire to inform themselves on the present state of this important department of science.

The translators have faithfully performed their duty, closely yet intelligibly rendering into our vernacular, the author's researches and opinions. Their additions, also, are generally judicious, and add considerably to the value of the work. If disposed to be critical, we might object to the occasional introduction of notes at variance with the text. Such liberties can only be sanctioned by an author's permission, and we have reason to know that in these instances Prof. Kölliker still maintains his own views.

The notes of the American Editor are always pertinent and consist mainly of his own pathological researches; of some recently described facts and opinions of the author; extracts from his former and larger work and of additional sources of reference. The publishers have issued the work in excellent style. Paper, print and illustrations are everything that could be wished.

---

*The Principles of Animal and Vegetable Physiology. A Popular Treatise on the Functions and Phenomena of Organic Life. To which is prefixed a General View of the Great Departments of Human Knowledge.* By STEVENSON BUSHNAN, M. D., *Physician to the Metropolitan Free Hospital, &c.*, Philadelphia. Blanchard & Lea, 1854.

The above little volume is the first of a series of treatises, published in London, under the title of "Orr's Circle of the Sciences." The author of it, Dr. Bushnan, is the editor of the series. Owen, Ansted, Latham, and others, are mentioned as original contributors to this enterprise, which has for its object the popularization of science.

The Introductory part of the present volume, attempts "to expound, in brief and lucid terms, the general nature, relations and application of all the chief departments of human knowledge, in order to give the reader, not specially trained in science, a general view of the vast field of enquiry which the Creator has laid open to the lawful exercise of the human intellect."

Forty-four pages are occupied with this exposition. This compass is entirely too narrow for the subject. It is obviously impossible that any attempt to comprise, in so brief a space, the multiform phenomena of mind and matter, however general the description may be, must prove a failure. Superficiality has always been the result of all such endeavors, and the present introduction only adds another example to the truth of the statement.

The second and principal part of the work is devoted to the

"Physiology of Animal and Vegetable Life." It gives us pleasure to commend this summary. It is clearly and concisely written, and accords with the views of the latest and best systematic treatises on the subject.

The work is largely illustrated.

---

*Healthy Skin: a Popular Treatise on the Skin and Hair—their Preservation and Management.* By ERASMUS WILSON, F. R. S., &c. *Second American, from the Fourth and Revised London Edition. With Illustrations.* Philadelphia, Blanchard & Lea. 1854.

We can cordially recommend the above little treatise as a excellent popular exposition of the subjects of which it treats. Much useful information to the practitioner of medicine will be found scattered throughout its pages. The important bearing of the skin and hair upon the general health of the system is strongly dwelt upon. The author has succeeded in making it an agreeable work, notwithstanding its scientific character—a a great merit in a popular treatise—the usefulness of all such being greatly enhanced when amusement is blended with instruction.

